# Regulating the active species of Ni(OH)_2_ using CeO_2_: 3D CeO_2_/Ni(OH)_2_/carbon foam as an efficient electrode for the oxygen evolution reaction†
†Electronic supplementary information (ESI) available: Full experimental procedures, experimental section, XPS spectra, TEM images, FTIR spectra and other electrochemical performance data. See DOI: 10.1039/c6sc05408k


**DOI:** 10.1039/c6sc05408k

**Published:** 2017-02-14

**Authors:** Zhengqing Liu, Na Li, Hongyang Zhao, Yi Zhang, Yunhui Huang, Zongyou Yin, Yaping Du

**Affiliations:** a Frontier Institute of Science and Technology Jointly with College of Science , State Key Laboratory for Mechanical Behavior of Materials , Xi’an Jiaotong University , 99 Yanxiang Road, Yanta District , Xi’an , Shaanxi Province 710054 , China . Email: ypdu2013@mail.xjtu.edu.cn; b Collaborative Innovation Center of Intelligent New Energy Vehicle , School of Materials Science and Engineering , Tongji University , Shanghai 201804 , P. R. China; c State Key Laboratory of Material Processing and Die & Mould Technology , School of Materials Science and Engineering , Huazhong University of Science and Technology , Wuhan , Hubei 430074 , P. R. China; d Research School of Chemistry , The Australian National University , Canberra , Australian Capital Territory 2601 , Australia; e Department of Materials Science and Engineering , Massachusetts Institute of Technology , Cambridge , MA 02139 , USA

## Abstract

Three dimensional (3D) N, O and S doped carbon foam (NOSCF) is prepared as a substrate for *in situ* vertically grown Ni(OH)_2_ nanosheets. As designed Ni(OH)_2_/NOSCF possesses strong electrostatic interactions with OH^–^ ions due to many C=O groups existing in NOSCF, which can facilitate the formation of crucial NiOOH intermediates during the OER process. CeO_2_ nanoparticles (NPs) of ∼3.3 nm in size are decorated on Ni(OH)_2_ nanosheets to design a highly efficient CeO_2_/Ni(OH)_2_/NOSCF electrocatalyst for the oxygen evolution reaction (OER). The CeO_2_ NP decorated Ni(OH)_2_/NOSCF not only exhibits a remarkably improved OER performance with an onset potential of 240 mV, outperforming most reported non-noble metal based OER electrocatalysts, but also possesses a small Tafel slope of 57 mV dec^–1^ and excellent stability under different overpotentials. The synergistic effect of producing more active species of Ni^III/IV^ and accelerating the charge transfer for Ni(OH)_2_/NOSCF by the introduction of CeO_2_ NPs is also investigated. These results demonstrate the possibility of designing energy efficient OER catalysts with the assistance of earth abundant CeO_2_-based catalysts.

## Abstract 2

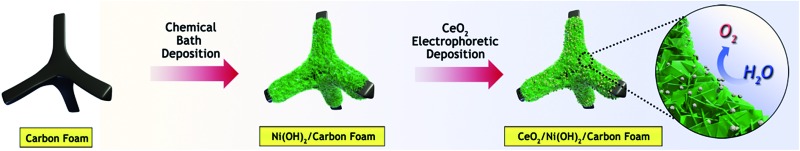
We found that Ni(OH)_2_ nanosheets of Ni(OH)_2_/NOSCF decorated with ∼3.3 nm CeO_2_ NPs displayed enhanced OER performance.

## Introduction

Clearly, using electricity to split water into hydrogen and oxygen (2H_2_O → 2H_2_ + O_2_) is one of the most efficient and attractive methods for the production of renewable energy.^1,2^ However, large-scale water electrolysis is greatly hindered due to the huge overpotential and significant efficiency loss for the half-cell of the oxygen evolution reaction (OER).^3–5^ Although noble-metal based materials (*e.g.* Ir, Pt) are currently regarded as high-efficiency OER catalysts, their low earth abundance and high cost limit their widespread use.^6–8^ Therefore, in recent years, various efficient and low-cost OER electrocatalysts (such as, Fe, Co, Ni and Mn) with high OER performance (low onset potential, high activity and good stability) in basic electrolytes have been extensively designed and investigated.^8–23^ Among them, nickel(ii) hydroxide (Ni(OH)_2_)-based materials are attractive electrocatalysts for the OER because of their intrinsic potential for high OER performance and two-dimensional (2D) layered structure.^20^ Another key reason for the extensive study of Ni(OH)_2_ is that the high oxidation state of Ni^III/IV^ can serve as an active species for OER catalysts.^24^ For example, Ye *et al.* prepared a Ni(OH)_2_–Au hybrid as an OER catalyst, and a significantly enhanced OER performance was exhibited by enhancing the generation of the Ni^III/IV^ active species. However, the poor kinetics and mass-transferability of Ni(OH)_2_ as an electrocatalyst for the OER still limit its further development for practical applications.

Cerium(iv) oxide (CeO_2_) is one of the most important rare earth oxides, is stable in alkaline solution, converts easily between the Ce^3+^ and Ce^4+^ oxidation states, undergoes reversible oxygen ion exchange (1/2O_2_ (gas) + 2e^–^ (solid) ↔ O^2–^ (solid)), and has good ionic conductivity and high oxygen-storage capacity (OSC).^25–27^ The above unique properties enable CeO_2_ to serve as a cocatalyst to enhance the performance of OER catalysts by improving charge transfer and energy conversion efficiency, which can also solve the poor kinetics and mass-transferability problems of Ni(OH)_2_ for the OER. However, few studies have focused on the application of CeO_2_ nanocrystals in the electrocatalytic field. Recently, Li *et al.* developed an efficient OER electrocatalyst by supporting FeOOH/CeO_2_ on Ni foam and exhibited enhanced OER performance compared with pure FeOOH.^28^ They also demonstrated the unique high OSC properties of CeO_2_, such that CeO_2_ can straightway absorb the oxygen produced during the OER and accordingly promote the OER. Therefore, the combination of Ni(OH)_2_ and CeO_2_ to form a CeO_2_/Ni(OH)_2_ hybrid will be an efficient route to improve the electrocatalytic performance of Ni(OH)_2_ *via* improving the energy conversion efficiency, and thereby promoting the generation of active species of Ni^III/IV^ for enhancing the OER performance.

In order to greatly prevent the Ni(OH)_2_ nanosheets from aggregation and thus further enhance the OER performance, three dimensional (3D) free-standing carbon foam (CF) is chosen as the substrate for *in situ* growth of the Ni(OH)_2_ nanosheets. The advantages of applying such 3D CF as a substrate can be attributed to the interconnected frameworks with large surface area for effective contact with an aqueous electrolyte and rapid interfacial electron charge transfer. Moreover, the obtained CF is doped by N, O and S elements during carbonization without other extra chemicals being added, where the N, O and S elements come from the melamine resin and sodium bisulfite additive of melamine foam (MF) (Fig. S1, ESI†). And N, O and S doped carbon materials are believed to enhance the OER activity.^29^

**Fig. 1:**
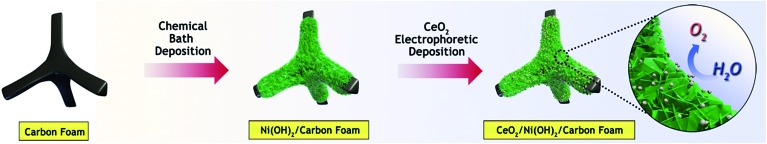
Process for the design of a self-supported CeO_2_/Ni(OH)_2_/NOSCF electrode and application for the oxygen evolution reaction.

Herein, as we expect, Ni(OH)_2_ nanosheets are successfully grown along the frameworks of N, O and S doped CF (NOSCF) and prevent the undesirable aggregation of Ni(OH)_2_ nanosheets because of the open cell pores of NOSCF. Then, we prepared uniform CeO_2_ NPs of ∼3.3 nm in size *via* a one step colloidal synthesis method, and deposited the surface modified-CeO_2_ NPs on Ni(OH)_2_ nanosheets of the as-designed Ni(OH)_2_/NOSCF to form a self-supported CeO_2_/Ni(OH)_2_/NOSCF electrode, as shown in Fig. 1. As a result of the open cell structure of 3D NOSCF for facile electrolyte transport and strong electronic interactions between CeO_2_ NPs and Ni(OH)_2_ nanosheets for accelerating the oxidation of Ni^II^ to Ni^III/IV^, the CeO_2_/Ni(OH)_2_/NOSCF electrocatalyst delivers an excellent water oxidation performance at a lower onset potential, ranking high among the extensive non-noble electrocatalysts studied for the OER. As we know, this is the first time CeO_2_ is combined with a functional Ni(OH)_2_ electrocatalyst, which offers an impressive OER performance, and provides insight into the possibility of enhancing OER catalysis by using rare earth CeO_2_-based nanomaterials.

## Results and discussion

### Design of the CeO_2_/Ni(OH)_2_/NOSCF electrocatalyst

**Fig. 2:**
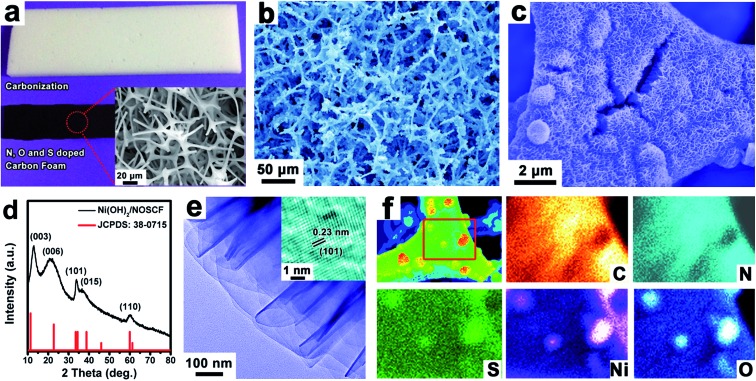
(a) A piece of melamine foam derived carbon foam. (b and c) SEM images of Ni(OH)_2_ nanosheets grown on NOSCF and the corresponding (d) XRD pattern. (e) HRTEM image of synthesized Ni(OH)_2_ recorded from the edge of the Ni(OH)_2_/NOSCF. (f) EDS elemental mapping for C, N, S, Ni and O elements recorded on the Ni(OH)_2_/NOSCF.

N, O and S doped CF was prepared by direct carbonization of melamine foam (MF) in a tube furnace at 700 °C for 1 h under protection of a nitrogen atmosphere. After carbonization, the volume of CF shrunk to about 70% of MF (inset of Fig. 2a). Then, the NOSCF was further oxidized by dipping into a mixture of acids of HNO_3_ (65%) and H_2_SO_4_ (98%) with a volume ratio of 1 : 3 for 6 h, which can produce more C=O groups (Fig. S2a, ESI†).^29^ It has been revealed that the C atoms in the C=O groups have a stronger electrostatic interaction with OH^–^ ions than other oxygen-containing groups, which can facilitate the formation of crucial M–OOH intermediates (eqn (1)), and thus accelerate the OER reaction.^30,31^

1

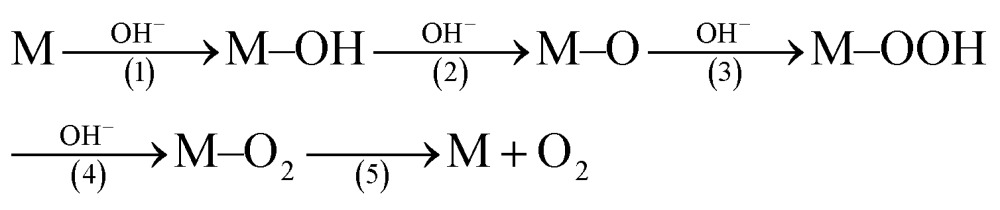




The elemental content in the NOSCF was measured by XPS analysis to be about 66.8, 4.3, 24.5 and 0.42 atom% for C, N, O and S, respectively (Fig. S2b, ESI†). Scanning electron microscopy (SEM) images in the inset of Fig. 2a revealed that the as-prepared NOSCF possessed an interconnected network architecture, which could make it an ideal substrate for the growth of some electrocatalysts. Then, by using the framework of NOSCF as a nucleation platform, Ni(OH)_2_ nanosheets could be uniformly grown *in situ* along the framework of NOSCF by a simple chemical bath deposition process,^32^ which could be observed evidently from the SEM images (Fig. 2b and c). The selective growth of Ni(OH)_2_ nanosheets on the NOSCF could preserve the open-cell structure of the NOSCF (Fig. 2b) and efficiently prevent the aggregation of Ni(OH)_2_ nanosheets, indicating that it held a large surface area for electrocatalysis. The vertical Ni(OH)_2_ layers could be clearly observed in an enlarged SEM image of the Ni(OH)_2_/carbon foam hybrid (Fig. S3, ESI†). Such nanostructured materials can offer a much rougher surface, which reduces the solid–gas interaction, giving rise to a timely release of adhered gas bubbles and thus enhancing the OER performance.

The grown Ni(OH)_2_ nanosheets had a hexagonal phase (*a* = *b* = 0.308 nm, *c* = 0.234, JCPDS: 38-0715), as confirmed by powder X-ray diffraction (PXRD) analysis (Fig. 2d). As shown in the transmission electron microscopy (TEM) image in Fig. 2e, the grown Ni(OH)_2_ presented a typical layered structure, and the high resolution (HRTEM) image (inset of Fig. 2e) identified the (101) plane of a hexagonal crystal structure for the Ni(OH)_2_ nanosheets with an interplanar spacing of 0.23 nm. The corresponding elemental mapping of the designed Ni(OH)_2_/NOSCF is shown in Fig. 2f; the C, N and S elements were distributed on the whole surface of the frameworks in the NOSCF, and also displayed a very uniform distribution of Ni(OH)_2_. The loading percentage of Ni(OH)_2_ in the Ni(OH)_2_/NOSCF composite was estimated to be ∼63% by thermogravimetric analysis (TGA), as displayed in Fig. S4 (ESI†).

**Fig. 3:**
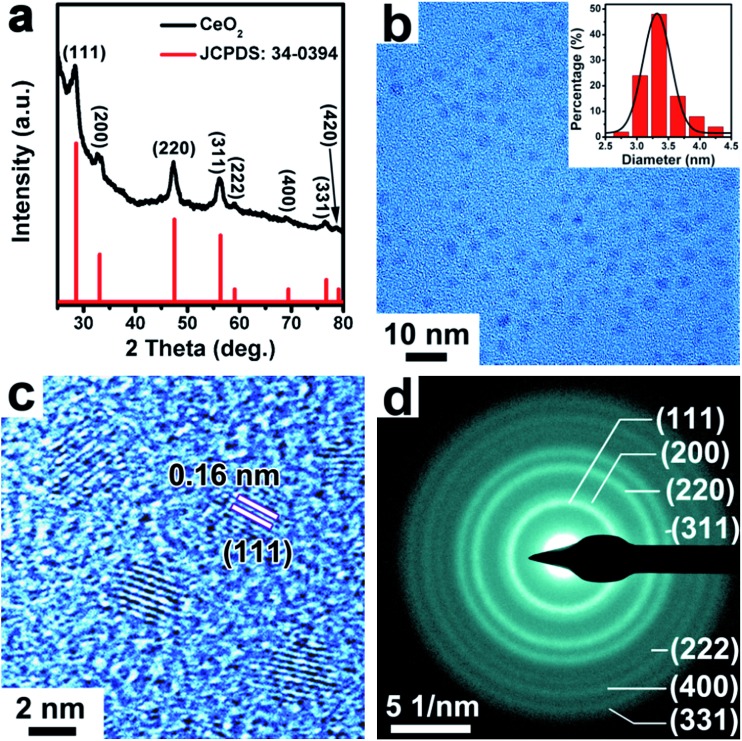
(a) XRD pattern of the obtained CeO_2_ sample. (b) TEM, (c) HRTEM image and (d) corresponding SAED pattern of synthesized ∼3.3 nm sized CeO_2_ NPs. Inset of (b): a particle size distribution analysis.

The CeO_2_ NPs were synthesized by using cerium(iv) ammonium nitrate ((NH_4_)_2_Ce(NO_3_)_6_) as a precursor in a mixture of solvents of oleylamine and 1-octadecene. As shown in the PXRD pattern in Fig. 3a, the prepared CeO_2_ samples presented a cubic phase (space group: *Fm*3*m*, *a* = *b* = *c* = 5.411 Å, JCPDS: 34-0394). The TEM image in Fig. 3b showed that the as-synthesized CeO_2_ NPs were relatively monodisperse with an average size of ∼3.3 nm (inset of a histogram of the particle diameters). The good monodispersity of the CeO_2_ NPs indicates the retention of the used capping ligand (oleylamine) on the surface of CeO_2_ NPs, as demonstrated by Fourier transform infrared (FTIR) spectroscopy (Fig. S5, ESI†). As seen in Fig. 3c, the HRTEM image of the CeO_2_ NPs showed clearly crystal lattice fringes with an interplanar spacing of 0.16 nm, which can be ascribed to the (111) crystal plane. The selected area electron diffraction (SAED) pattern shown in Fig. 3d indicated that the synthesized CeO_2_ NPs were highly crystallized.

**Fig. 4:**
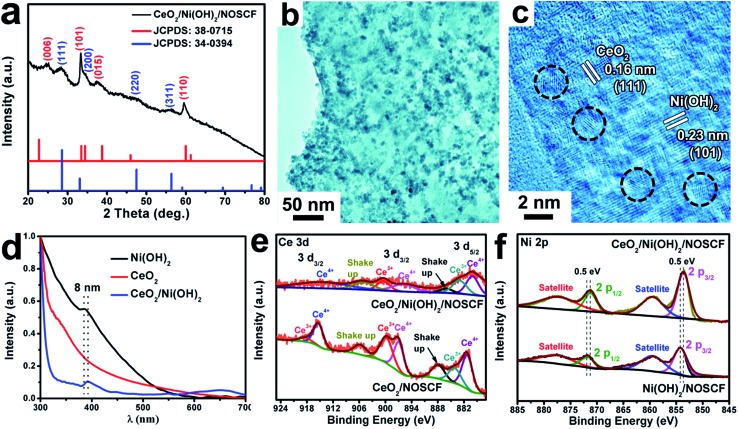
(a) XRD pattern, (b) TEM and (c) HRTEM images of CeO_2_/Ni(OH)_2_ hybrid nanostructures obtained from the CeO_2_/Ni(OH)_2_/NOSCF. (d) UV-vis absorption spectra of pristine Ni(OH)_2_ nanosheets, CeO_2_ NPs and the CeO_2_/Ni(OH)_2_ hybrid. A comparison of (e) Ce 3d and (f) Ni 2p high-resolution XPS spectra of CeO_2_/Ni(OH)_2_/NOSCF and Ni(OH)_2_/NOSCF.

The prepared CeO_2_ NPs are hydrophobic due to the long carbon chains of oleylamine (OM) used as surfactants for the reaction, and hence cannot directly disperse in water. In order to generate a hydrophilic surface for combining with the Ni(OH)_2_/NOSCF and testing the OER performance, we employed NaS_2_ solution to modify the surface of CeO_2_ NPs (Fig. S5, ESI†).^33^ As shown in the TEM image in Fig. S6a (ESI†), the CeO_2_ NPs still kept their particle morphology with high crystallization after surface modification, and could be well dispersed in water (digital photo in Fig. S6b, ESI†). The CeO_2_ NPs were anchored on the Ni(OH)_2_/NOSCF using a controllable electrophoretic deposition strategy, the details are shown in the Experimental section.^34^ All of the diffraction peaks of Ni(OH)_2_ (JCPDS: 380715) and CeO_2_ (JCPDS: 34-0394) were detected in CeO_2_/Ni(OH)_2_/NOSCF (Fig. 4a). Fig. 4b and c show the representative TEM and HRTEM images of the CeO_2_/Ni(OH)_2_ hybrid obtained from CeO_2_/Ni(OH)_2_/NOSCF with a deposition duration of 10 min. It can be observed from Fig. 4b that the Ni(OH)_2_ nanosheets are uniformly decorated with CeO_2_ NPs. Fig. 4c presents the corresponding HRTEM image with an interplanar spacing of 0.16 nm and 0.23 nm, indexed to the (111) and (101) crystal planes of CeO_2_ and Ni(OH)_2_, respectively.

To further investigate the strong electronic interactions between Ni(OH)_2_ nanosheets and CeO_2_ NPs, UV-vis absorption spectra (Fig. 4d) and X-ray photoelectron spectroscopy (XPS) spectra (Fig. 4e and f) of Ni(OH)_2_ nanosheets, CeO_2_ NPs and the CeO_2_/Ni(OH)_2_ hybrid were examined. As shown in Fig. 4d, two absorption peaks of the grown Ni(OH)_2_ nanosheets located at 385 and 670 nm corresponded to the d–d transitions of Ni^II^ cations.^35^ Compared with the pristine Ni(OH)_2_ nanosheets, the absorption spectrum of CeO_2_/Ni(OH)_2_ was obviously red-shifted (∼8 nm), indicating the strong electronic interactions between them.^36,37^ XPS spectra of Ce 3d and Ni 2p are shown in Fig. 4e and f, respectively. As shown in Fig. 4e, for Ce 3d of CeO_2_, the peaks located at 920–911 eV and 903–893 eV correspond to Ce 3d_3/2_, and the peaks located at 877–866 eV correspond to Ce 3d_5/2_, which demonstrated the coexistence of Ce^3+^ and Ce^4+^ in the CeO_2_ NPs.^38^ However, after CeO_2_ NPs were deposited on the Ni(OH)_2_ nanosheets, the ratio of Ce^3+^ : Ce^4+^ in the CeO_2_/Ni(OH)_2_ hybrid changed compared with pure CeO_2_ NPs, indicating that the valence states of Ce in the CeO_2_/Ni(OH)_2_ hybrid rearranged.^28^ As shown in Fig. 4f, the XPS spectrum of Ni 2p in Ni(OH)_2_/NOSCF showed two major peaks at 853.2 and 870.8 eV corresponding to Ni 2p_3/2_ and Ni 2p_1/2_, respectively, which were characteristic of the Ni^2+^ state.^39^ And some satellite peaks in the Ni 2p region could also be observed in Fig. 4f. Through careful comparison and analysis, we found that the peaks of Ni 2p_3/2_ and Ni 2p_1/2_ in the XPS spectrum for CeO_2_/Ni(OH)_2_/NOSCF both shifted to lower binding energies of ∼0.5 eV. Therefore, the ratio change of Ce 3d and peak shifts of Ni 2p in the CeO_2_/Ni(OH)_2_ hybrid indicate strong electronic interactions between the Ni(OH)_2_ nanosheets and CeO_2_ NPs.

### CeO_2_/Ni(OH)_2_/NOSCF electrocatalyst for OER performance

**Fig. 5:**
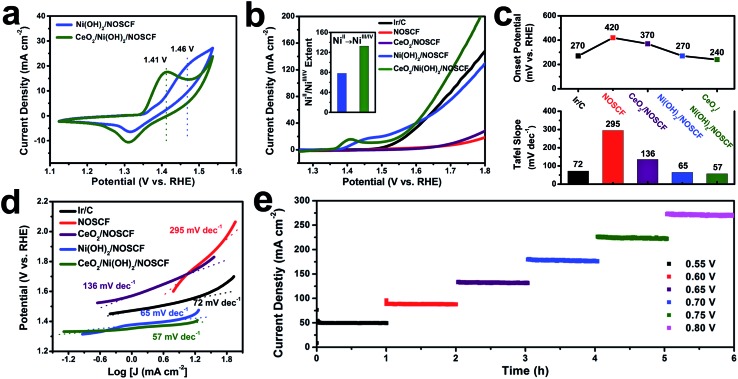
(a) CV curves of Ni(OH)_2_/NOSCF and CeO_2_/Ni(OH)_2_/NOSCF. (b) Polarization curves of NOSCF, CeO_2_/NOSCF, Ni(OH)_2_/NOSCF, CeO_2_/Ni(OH)_2_/NOSCF and the benchmark Ir/C electrocatalyst for comparison. Sweep rate: 5 mV s^–1^. Inset: the extent of Ni^II^/Ni^III/IV^ transformation for Ni(OH)_2_/NOSCF and CeO_2_/Ni(OH)_2_/NOSCF. (c and d) The corresponding onset potential and Tafel curves for the catalysts derived from (b). (e) Stability test using a continuous OER recorded on the CeO_2_/Ni(OH)_2_/NOSCF self-supported electrode under different static potentials (V *vs.* SCE).

The water oxidation reaction was applied to study the electronic interactions between Ni(OH)_2_ nanosheets and CeO_2_ NPs and their effect on the corresponding catalytic activity. The OER performance of the CeO_2_/Ni(OH)_2_/NOSCF electrocatalyst was investigated in 1.0 M KOH (pH = 14). As a comparison, Ni(OH)_2_/NOSCF, CeO_2_/NOSCF, NOSCF and Ir/C were also tested under the same conditions. It was obvious from the cyclic voltammetry (CV) curves (Fig. 5a) that both the electrocatalysts of Ni(OH)_2_/NOSCF and CeO_2_/Ni(OH)_2_/NOSCF clearly showed redox peaks ranging from 1.2 to 1.6 V, which belong to the Ni^II^/Ni^III/IV^ redox process (Ni(OH)_2_ + OH^–^ → NiOOH + H_2_O + e^–^). And there was an obvious negative shift of the oxidation potential, changed from 1.46 V to 1.41 V for CeO_2_/Ni(OH)_2_/NOSCF, indicating that CeO_2_/Ni(OH)_2_/NOSCF has higher transfer efficiency from Ni^II^ to Ni^III/IV^ and larger charge capacity than Ni(OH)_2_/NOSCF.^40^ This conclusion can be further demonstrated by Nyquist plots. As presented in Fig. S7 (ESI†), compared with Ni(OH)_2_/NOSCF, the CeO_2_/Ni(OH)_2_/NOSCF shows a smaller charge transfer resistance (high frequencies) and reduced mass-transfer resistance (low frequencies). The decrease in mass transfer resistance may contribute to the increased number of Ni^III/IV^ active species.

As shown in the polarization curves in Fig. 5b and statistical data in Fig. 5c, the CeO_2_/Ni(OH)_2_/NOSCF exhibited a lower onset potential of 240 mV than Ir/C and Ni(OH)_2_/NOSCF, surpassing most reported non-noble metal based OER electrocatalysts (Table S1, ESI†). In particular, the significant increase of the current density was more obvious when the potential was beyond ∼1.6 V, which could further demonstrate that the OER activity of Ni(OH)_2_/NOSCF is greatly enhanced when decorated with CeO_2_ NPs. In addition, we also optimized the loaded mass ratio of CeO_2_ NPs on Ni(OH)_2_/NOSCF and found that when the mass ratio of CeO_2_ : Ni(OH)_2_/NOSCF was 30% (Fig. S8, ESI†), the electrocatalytic activity of CeO_2_/Ni(OH)_2_/NOSCF reached its highest level, and therefore this mass ratio was used in the following experiments.

During the OER process, the highly oxidative Ni^III/IV^ cations are believed to serve as active species, which indicates that the enhanced catalytic activity for CeO_2_/Ni(OH)_2_/NOSCF observed in our study might be a result of increasing Ni^II^/Ni^III/IV^ transformations. Therefore, we investigated the extent of the Ni^II^/Ni^III/IV^ transformation by integrated oxidation peak areas (inset of Fig. 5b).^41,42^ When CeO_2_ NPs were deposited on the Ni(OH)_2_/NOSCF, the Ni^II^/Ni^III/IV^ extent showed a dramatic increase of about 1.7-fold compared with the Ni(OH)_2_/NOSCF (inset of Fig. 5b), thus CeO_2_ NPs potentially facilitated producing more Ni^III/IV^ active species and subsequently led to the improvement of the OER catalytic activity (Fig. 5b).

The enhanced OER activity of CeO_2_/Ni(OH)_2_/NOSCF was more obvious by comparing the Tafel slopes. As shown in Fig. 5c and d, the Tafel slope of CeO_2_/Ni(OH)_2_/NOSCF was 57 mV dec^–1^, and it was smaller than those of Ir/C (72 mV dec^–1^), NOSCF (295 mV dec^–1^), CeO_2_/NOSCF (136 mV dec^–1^), and Ni(OH)_2_/NOSCF (65 mV dec^–1^). Through the comparison of the Tafel slopes we could demonstrate that depositing CeO_2_ NPs on Ni(OH)_2_/NOSCF could facilitate its OER kinetics, and the OER activity of CeO_2_/Ni(OH)_2_/NOSCF was comparable to many other non-noble metal OER electrocatalysts in alkaline media (Table S1, ESI†).

We also tested the stability of the designed CeO_2_/Ni(OH)_2_/NOSCF by a chronoamperometry method to evaluate the OER performance. As shown in Fig. 5e, the current density of the OER showed no change during 6 h of continuous operation under various potentials of 0.55, 0.60, 0.65, 0.70, 0.75 and 0.80 V, which suggested that the CeO_2_/Ni(OH)_2_/NOSCF had excellent stability for the OER process. Thus, the CeO_2_/Ni(OH)_2_/NOSCF with its high catalytic activity as well as excellent stability would be a promising candidate for electrochemical water oxidation.

## Conclusions

In summary, we have successfully designed a 3D hierarchical Ni(OH)_2_/NOSCF electrode by growing Ni(OH)_2_ nanosheets along the carbon frameworks of N, O and S doped CF. The experiments found that Ni(OH)_2_ nanosheets of Ni(OH)_2_/NOSCF decorated with ∼3.3 nm sized CeO_2_ NPs displayed enhanced OER performance. Compared with Ni(OH)_2_/NOSCF, the onset potential of CeO_2_ NP decorated Ni(OH)_2_/NOSCF decreased from 270 to 240 mV, and the Tafel slope reduced from 65 to 57 mV dec^–1^, much better than the benchmark Ir/C. As confirmed by UV-vis and XPS results, as well as electrochemical analysis, the reasons for the enhanced OER performance result from the synergistic effect between CeO_2_ NPs and Ni(OH)_2_ nanosheets by a 1.7-fold enhancement in the generation of Ni^III/IV^ active species and faster charge transfer. The high OER performance of CeO_2_/Ni(OH)_2_/NOSCF in the present study makes CeO_2_ based composites very promising electrocatalysts for water oxidation.
