# Supplementary material for: Regulating the active species of Ni(OH)2 using CeO2: 3D CeO2/Ni(OH)2/carbon foam as an efficient electrode for the oxygen evolution reaction
Source: Chem Sci. 2017 Feb 14;8(4):3211–7. doi: 10.1039/c6sc05408k (PMC5414546; doi:10.1039/c6sc05408k)
Supplement: Supplementary file 1 [file SC-008-C6SC05408K-s001.pdf]

Electronic Supplementary Material (ESI) for Chemical Science.
This journal is © The Royal Society of Chemistry 2017

Electronic Supplementary Information (ESI)

Regulating Active Species of $Ni(OH)_2$ by CeO_2: 3D $CeO_2/Ni(OH)_2$/Carbon Foam as an Efficient Electrode for Oxygen Evolution Reaction

Zhengqing Liu, Na Li, Hongyang Zhao, Yi Zhang, Yunhui Huang, Zongyou Yin and Yaping Du

Experimental Section

*Preparation of N, O, S Doped **CF***: Firstly, commercial melamine foam (MF) was cut into small pieces. A piece of MF was put into a tube furnace and heated to 700 °C for 1 h under protection of nitrogen atmosphere. Then the as-prepared carbon foam (**CF**) was cooled down to room temperature. Secondly, a piece of as-prepared **CF** was immersed completely in the (4 mL 65% HNO_3 + 12 mL 98% H_2SO_4) mixed acid solution at 35 °C for 6 h. The obtained NOS**CF** was washed by deionized water till the pH of solution was neutral.

*Growth of $Ni(OH)_2$ Nanosheets on NOS**CF***: 5.26 g of $NiSO_4{\cdot}6H_2O$, 1 g of $K_2S_2O_8$ and 100 mL of deionized (DI) water (H_2O) were added in a 150 mL beaker at room temperature. A piece of as-prepared NOS**CF** was used as a substrate, and immersed into the above clear green solution. Then 3 mL of $NH_3{\cdot}H_2O$ solution was added drop by drop with stirring for a few seconds, let set for 1 h without stirring at room temperature.

Colloidal Synthesis of Monodispersed CeO_2 NPs: The 3.3 nm-sized CeO_2 NPs was prepared by one pot colloidal synthesis method. 2 mmol (109.6 mg) of $(NH_4)_2Ce(NO_3)_6$, 20 mmol (5.36 g) of OM and 20 mmol (5.05 g) of ODE were added into a 100 mL three-necked flask, removed water and oxygen by heating up to 120 °C. Then the resulting slurry was heated to designed reaction temperature of 300 °C under nitrogen atmosphere, and reacted for 40 min. After cooling to room temperature, the CeO_2 product was precipitated by excess ethanol and separated by centrifugation (10,000 rpm, 8 min), followed by dispersed in 5 mL hexane and removed long-chain ligand by adding 5 mL Na_2S (50 mg mL^{-1}) solution.

*Decoration of CeO_2 NPs on $Ni(OH)_2$/NOS**CF** to Form the $CeO_2/Ni(OH)_2$/NOS**CF** Electrode*: Electrophoretic deposition method was used for depositing CeO_2 NPs on $Ni(OH)_2$/NOS**CF**. Firstly,

the surfaced modified CeO_2 NPs were re-dispersed in acetone with various concentrations. The electrophoretic deposition process was performed with a two-electrode system. $Ni(OH)_2$/NOS**CF** was used as working electrode and Pt foil was used as counter electrode in the CeO_2 NPs dispersion. A constant voltage of 100 V/cm was applied for 10 mins.

Characterizations: The shapes of NOS**CF** and $Ni(OH)_2$/NOS**CF** were examined through a Quanta F250 scanning electron microscope (SEM). The morphologies of CeO_2 were characterized by a Hitachi HT−7700 transmission electron microscope (TEM, Japan), operating at 100 kV. High−resolution TEM (HRTEM) images were obtained by a Philips Tecnai F20 FEG−TEM (USA), operating at 200 kV. The podwer X-ray diffraction (XRD) patterns were obtained using a Rigaku D/MAX−RB with Cu Kα radiation (λ=1.5418 Å). FT-IR spectra were measured by a Nicolet 6700 FT-IR spectrometer. X-ray photoelectron spectra (XPS) were conducted by a PHI Quantera SXM spectrometer. UV-vis absorption spectra were measured by a PerkinElmer Lambda 35 UV−vis spectrophotometer.

Electrocatalytic Measurements for OER: The electrochemical measurements were carried out in an O_2 -saturated solution of 1.0 M KOH on an AUTOLAB PGSTAT204 electrochemistry workstation by using a typical three-electrode electrochemical cell. A saturated calomel electrode (Hg/$HgCl_2$ in saturated KCl) and Pt plate were used as the reference electrode and the counter electrode, respectively. The as prepared CeO_2/$Ni(OH)_2$/NOS**CF** directly served as working electrode. CV and LSV curves with a scan rate of 10 and 5 mV s^{-1} were conducted, respectively. EIS results were recoreded in the frequency range from 1.0 to 100 kHz at an open circuit potential with a 5 mV amplitude. Stability of CeO_2/$Ni(OH)_2$/NOS**CF** was conducted by chronoamperometry method under different overpotentials (vs. SCE) of 0.55, 0.60, 0.65, 0.70, 0.75, 0.80 V for continuous testing of 6 h.

Electronic Supplementary Information (ESI)

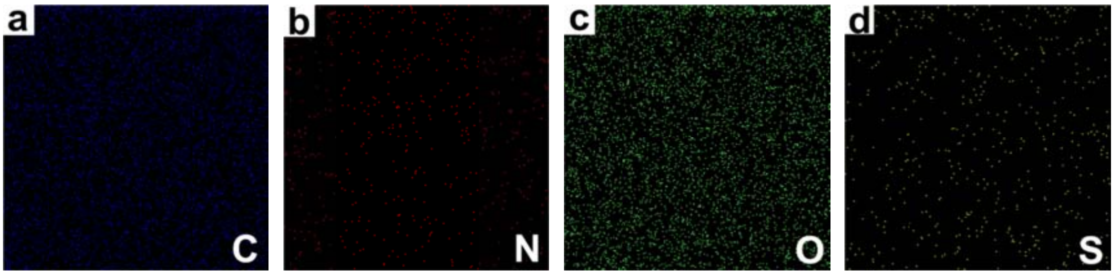

Fig. S1 EDS elemental mapping for C, N, O and S elements recorded on the pure carbon foam.

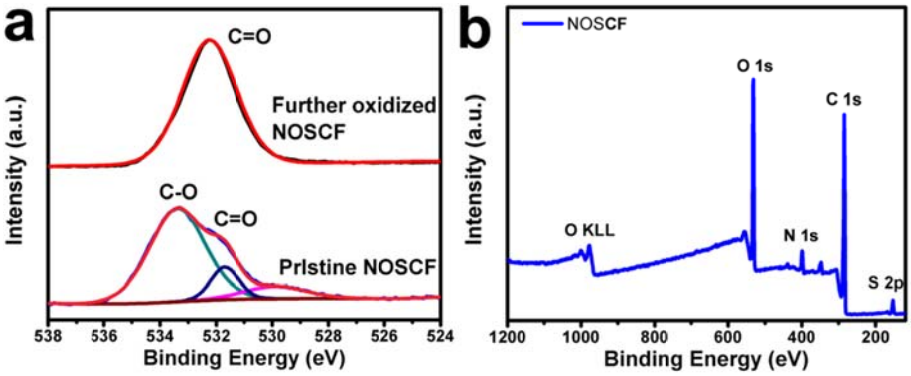

Fig. S2 (a) High-resolution O 1s XPS spectra of NOS**CF** and further oxidized NOS**CF**. (b) XPS spectrum of N, O and S doped carbon foam (NOS**CF**), the contents of C, N, O and S elements in NOS**CF** were measured to be about 66.8, 4.3, 24.5 and 0.42 atom %, respectively.

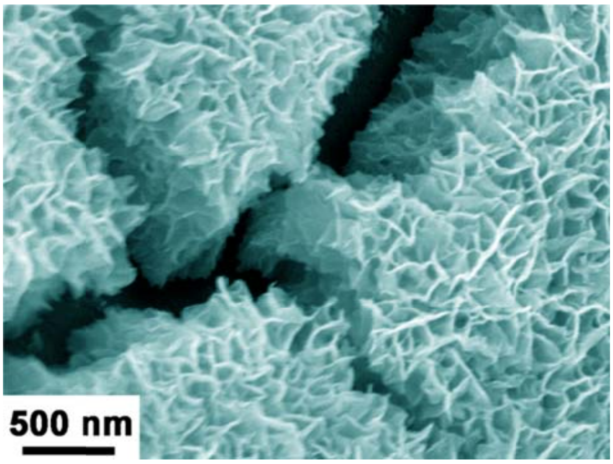

Fig. S3 The enlarged SEM image of $Ni(OH)_2$ nanosheets grown on NOS**CF.**

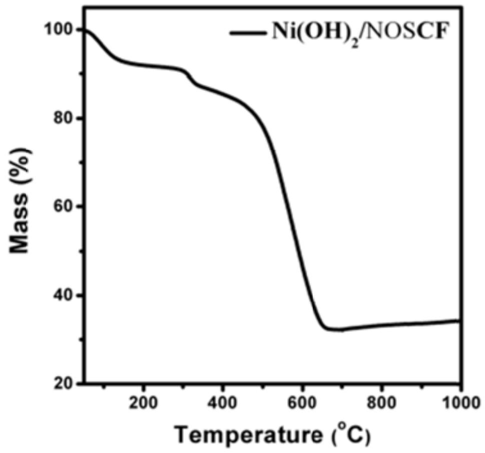

Fig. S4 The loading percentage of $Ni(OH)_2$ in the $Ni(OH)_2$/NOS**CF** was determined by thermogravimetric analysis (TGA). As shown in Fig. S2, the sample weight decreases slowly between 50 and 150°C; this is mainly due to the removal of chemically adsorbed H_2O. The slight weight loss of 3.75% between 300 and 350 °C is believed that the reaction of $Ni(OH)_2 \rightarrow NiO+H_2O$ occurs in this region. With continue to increase the temperature to more than 450 °C, an obvious weight loss of 50.8 % is present between 450 and 660 °C, indicating the NOS**CF** begins to convert into CO_2. Through analysis of the TGA data, the loading percentage of $Ni(OH)_2$ in the $Ni(OH)_2$/NOS**CF** composite was estimated to be ~63%.

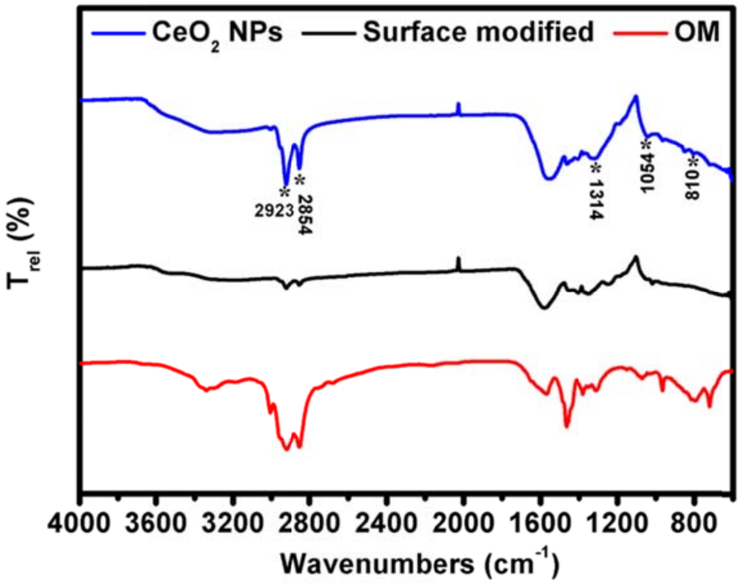

Fig. S5 FTIR spectra (Nicolet 6700) of oleylamine, as-prepared CeO_2 NPs and surface modified CeO_2 NPs. The presence of acyclic C-H stretching at 2923 cm^{-1} and 2854 cm^{-1} indicate the existence of oleylamine. The three sharp peaks at 1314, 1054 and 810 cm^{-1} in the IR spectrum of CeO_2 NPs were indexed to C–N stretch of oleylamine, respectively. Based on the FTIR analysis of CeO_2 NPs, it is confirmed that these CeO_2 NPs are coated by oleylamine organic molecule. Note here, after surface modified treatment, the intensity of characteristic peaks of oleylamine obviously decreased, indicating a successful removal of organic capping ligands from the surface of CeO_2 NPs.

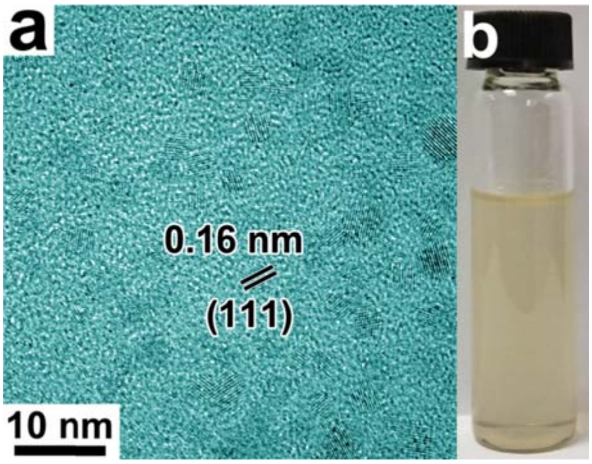

Fig. S6 (a) TEM image of CeO_2 NPs after surface modification. The interplanar spacing of 0.16 nm can be observed, which is corresponding to the (111) crystal plane of CeO_2 NPs. (b) Digital photo for surface modified-CeO_2 NPs dispersed in water solution.

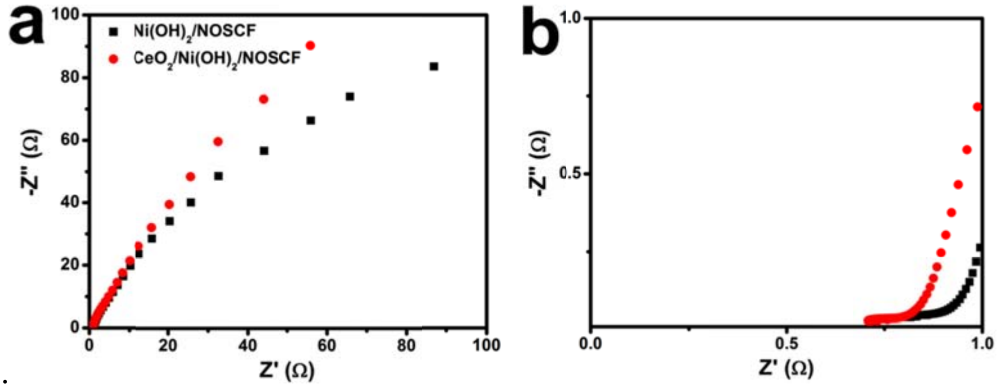

Fig. S7 (a) EIS Nyquist plots showing the electrode kinetics of $Ni(OH)_2$/NOS**CF** and $CeO_2/Ni(OH)_2$/NOS**CF**. (b) Enlarged EIS Nyquist plots derived from a).

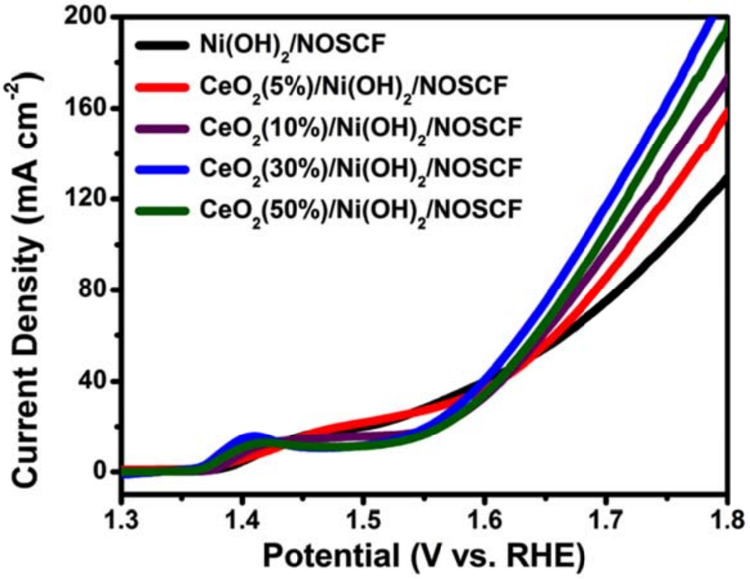

Fig. S8 Polarization curves recorded on CeO_2/$Ni(OH)_2$/NOS**CF** electrode with different content of CeO_2 NPs in CeO_2/$Ni(OH)_2$/NOS**CF**. The loading mass ratio of CeO_2 NPs on $Ni(OH)_2$/NOS**CF** were 5%, 10%, 30% and 50%, potential scan rate is 5 mV s^{-1}.

Table S1. Comparisons of the OER activity for CeO_2/$Ni(OH)_2$/NOS**CF** electrocatalyst with some representative OER catalysts under basic condition.

**Catalysts**	**Electrolyte**	**Onset potential**	**Tafel slope (mV dec^{-1})**	**Ref.**
**CeO_2/$Ni(OH)_2$/ NOSCF**	**KOH**	**240**	**57**	**Our work**
**CeO_2/FeOOH HLNTs-NF**	**KOH**	**220**	**92.3**	**S1**
**CeO_2/$CoSe_2$ nanobelts**	**KOH**	**270**	**44**	**S2**
**$Ni(OH)_2$-Au**	**KOH**	**240**	**36**	**S3**
**α-Nickel-Hydroxide**	**KOH**	**310**	**42**	**S4**
**Co-Mn LDHs**	**KOH**	**270**	**43**	**S5**
**Li-Ni-Co-OH**	**NaOH**	**-**	**67**	**S6**
**Fe-Ni LDHs**	**KOH**	**250**	**41**	**S7**

References:

S1. J. X. Feng, S. H. Ye, H. Xu, Y. X. Tong and G. R. Li, *Adv. Mater.*, 2016, **28**, 4698.
S2. Y. R. Zheng, M. R. Gao, Q. Gao, H. H. Li, J. Xu, Z. Y. Wu and S. H. Yu, *Small*, 2015, **11**, 182.
S3. G. G. Liu, P. Li, G. X. Zhao, X. Wang, J. T. Kong, H. M. Liu, H. B. Zhang, K. Chang, X. G. Meng, T. Kako and J. H. Ye, *J. Am. Chem. Soc.*, 2016, **138**, 9128.
S4. M. Gao, W. Sheng, Z. Zhuang, Q. Fang, S. Gu, J. Jiang and Y. Yan, *J. Am. Chem. Soc.,* 2014, **136**, 7077.
S5. F. Song and X. Hu, *J. Am. Chem. Soc.*, 2014, **136**, 16481.
S6. K. Y. Niu, F. Lin, S. Jung, L. Fang, D. Nordlund, C. C. L. McCrory, T. C. Weng, P. Ercius, M. M. Doeff and H. Zheng, *Nano Lett.*, 2015, **15**, 2498.
S7. C. Tang, H. S. Wang, H. F. Wang, Q. Zhang, G. L. Tian, J. Q. Nie and F. Wei, *Adv. Mater.*, 2015, **27**, 4516.
